# Clinical Valve Thrombosis and Subclinical Leaflet Thrombosis Following Transcatheter Aortic Valve Replacement: Is There a Need for a Patient-Tailored Antithrombotic Therapy?

**DOI:** 10.3389/fcvm.2019.00044

**Published:** 2019-04-18

**Authors:** Liesbeth Rosseel, Ole De Backer, Lars Søndergaard

**Affiliations:** The Heart Center, Rigshospitalet, Copenhagen, Denmark

**Keywords:** aortic valve replacement, transcatheter, clinical valve thrombosis, subclinical leaflet thrombosis, antithrombotic therapy

## Abstract

Transcatheter aortic valve replacement (TAVR) has become an established therapeutic option for patients with symptomatic, severe aortic valve stenosis at increased surgical risk. Antithrombotic therapy after TAVR aims to prevent transcatheter heart valve (THV) thrombosis, in which two different entities have to be recognized: clinical valve thrombosis and subclinical leaflet thrombosis. In clinical valve thrombosis, obstructive thrombus formation leads to an increased transvalvular gradient, often provoking heart failure symptoms. Subclinical leaflet thrombosis is most often an incidental finding, characterized by a thin layer of thrombus covering the aortic side of one or more leaflets; it is also referred to as Hypo-Attenuating Leaflet Thickening (HALT) as described on multi-detector computed tomography (MDCT) imaging. This phenomenon may also affect leaflet motion and is then classified as Hypo-Attenuation affecting Motion (HAM). Even in case of HAM, the transvalvular pressure gradient remains within normal range and does not provoke heart failure symptoms. Whereas, clinical valve thrombosis requires treatment, the clinical impact and need for intervention in subclinical leaflet thrombosis is still uncertain. Oral anticoagulant therapy protects against and resolves both clinical valve thrombosis and subclinical leaflet thrombosis; however, large-scale randomized clinical trials studying different antithrombotic strategies after TAVR are still under way. This review article summarizes the currently available data within the field of transcatheter aortic valve/leaflet thrombosis and discusses the need for a patient tailored antithrombotic approach.

## Introduction

Transcatheter aortic valve replacement (TAVR) has become an established therapeutic option for patients with symptomatic, severe aortic valve stenosis who are at increased surgical risk. Furthermore, the NOTION trial (Nordic Aortic Valve Intervention Trial) showed that TAVR may also be a viable option for patients with a lower risk profile (1). Antithrombotic therapy after TAVR aims to prevent transcatheter heart valve (THV) thrombosis. Two different entities have to be recognized: clinical valve thrombosis and subclinical leaflet thrombosis. This review aims to summarize and discuss currently available data on clinical valve thrombosis and subclinical leaflet thrombosis in transcatheter aortic bioprosthesis with additional focus on a patient-tailored antithrombotic approach.

## Definition and Incidence

Although prosthetic valve thrombosis is a well-known risk for mechanical heart valves, this risk is much less recognized in bioprosthetic heart valves. Following implantation of mechanical valve prostheses, patients have to take life-long oral anticoagulant (OAC) therapy. This is not the case for patients who receive a surgical or transcatheter bioprosthetic heart valve, for which long-term treatment with antiplatelet therapy (APT) seems to be sufficient. However, despite the less thrombogenic profile of bioprosthetic heart valves, recent studies also report the occurrence of valve/leaflet thrombosis in different types of transcatheter and surgical aortic bioprosthesis (2–10). Thereby, it is important to make the difference between two different entities: clinical valve thrombosis and subclinical leaflet thrombosis.

**Figure 1:**
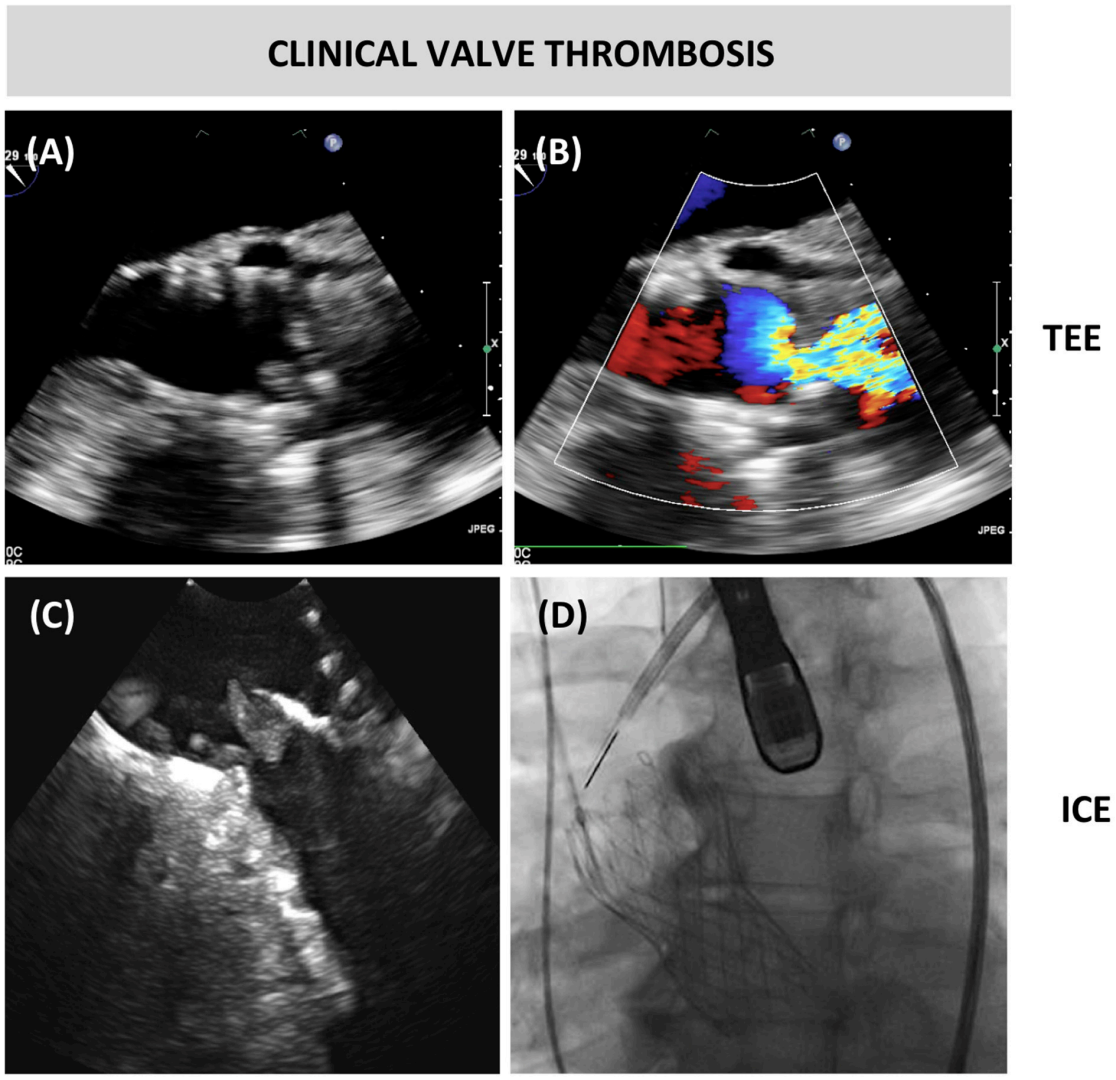
Clinical valve thrombosis. **(A,B)** Transesophageal echocardiography (TEE) showing thrombosis and turbulent color flow over a bioprosthetic aortic valve in a patient who underwent transcatheter aortic valve replacement 6 years earlier. The patient presented with dyspnea NYHA class 3b and echocardiography revealed a mean transvalvular gradient of 37 mmHg **(C,D)** The thrombotic mass at the prosthetic leaflets was confirmed by intracardiac echocardiography from the ascending aorta.

Clinical valve thrombosis is defined as clinical apparent prosthetic valve dysfunction with the typical finding of a mobile mass/thrombus on the prosthetic heart valve as visualized by echocardiography or multi-detector computed tomography (MDCT). Prosthetic valve dysfunction may be provoked by reduced leaflet motion or impaired leaflet coaptation caused by thrombus, but it is important that this gets differentiated from other causes such as valve degeneration, fibrotic pannus ingrowth or endocarditis (Figure 1). The clinical appearance of this phenomenon may be either symptoms of heart failure, or left sided thrombo-embolic event.

Following surgical aortic valve replacement (SAVR) with a bioprosthesis, the incidence of clinical valve thrombosis has been reported to range between 0.3 and 6.0% (11–14). More recently, this phenomenon of clinical valve thrombosis has also been described following TAVR. Based on retrospective observational registries, the incidence of clinical valve thrombosis in TAVR patients is estimated to be 0.6 to 2.8% (15, 16).

**Figure 2:**
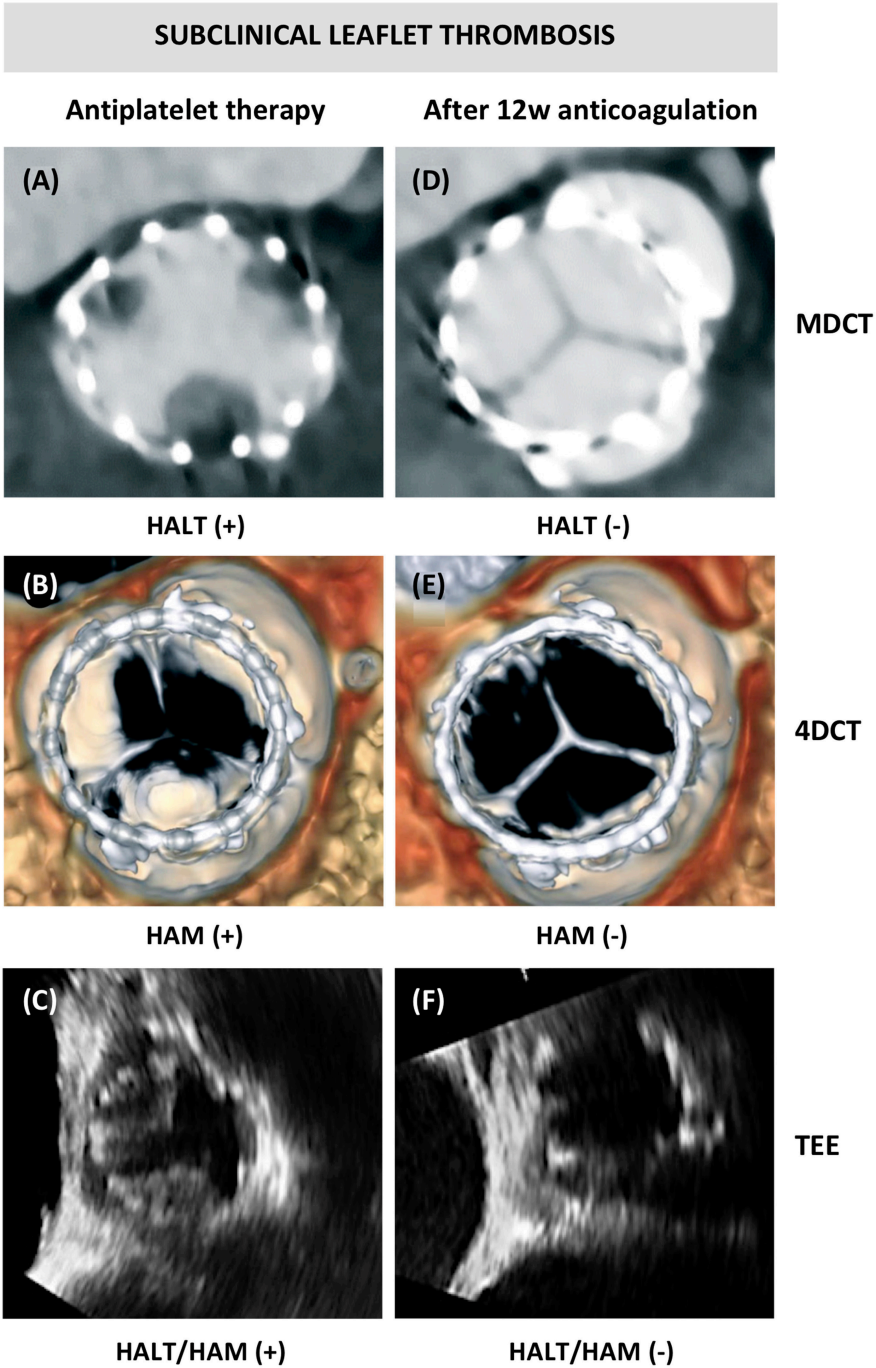
Subclinical leaflet thrombosis. **(A)** Computed tomography (CT) images showing hypoattenuating leaflet thickening (HALT) at the base of all three bioprosthetic leaflets, **(B)** with hypoattenuation affecting motion (HAM) visible in systole in the volume-rendered 4DCT images; and **(C)** transesophageal echocardiography (TEE) confirming reduced leaflet motion of two leaflets. **(D–F)** Resolution of HALT and HAM after 3 months of anticoagulation treatment.

Subclinical leaflet thrombosis was firstly described in a case report in 2013, 7 days after TAVR while doing routine MDCT imaging (17). A hypoattenuating structure in one cusp thereby restricting normal cusp movement was described, although a normal transvalvular pressure gradient was measured on transthoracic echocardiography (TTE). Control CT after 10 weeks OAC treatment showed full resolution of the initial finding. This phenomenon was than further investigated in the combined SAVORY/RESOLVE registry, describing the existence of leaflet thickening and reduced leaflet motion in different types of transcatheter and surgical aortic bioprosthesis (2). Subclinical leaflet thrombosis is described as a thin layer of thrombus that can involve one or all three leaflets, with typical appearance on MDCT as a hypo-attenuating defect at the aortic side of the leaflets, also called Hypo-Attenuating Leaflet Thickening (HALT). When leaflet motion is affected for more than 50%, this phenomenon is classified as Hypo-attenuation Affecting Motion (HAM) (Figure 2). As this leaflet thickening is typically an incidental finding not causing any clinically significant valvular dysfunction, it is called “subclinical” leaflet thrombosis.

The reported incidence of subclinical leaflet thrombosis is likely to be influenced by the timing and the intensity of screening as well as the applied diagnostic criteria and imaging tools. Table 1 gives an overview of available studies that performed prospective screening for subclinical leaflet thrombosis. Incidence of subclinical leaflet thrombosis, with or without reduced leaflet motion, varies between 7 and 35% in different types of THVs (2–9). Only few studies report on the incidence of subclinical leaflet thrombosis in surgical aortic bioprosthesis. In the SAVORY/RESOLVE registry, the incidence of subclinical leaflet thrombosis in surgical aortic bioprosthesis appeared to be lower than in THVs with an incidence rate of 4%, although SAVR patients were younger and with less comorbidities than the TAVR population (5). Finally, in a prospective trial investigating a sutureless type of surgical aortic bioprosthesis, a higher incidence was reported with 38% of patients having HALT and 28% showing HAM (10).

**Table 1:** Overview of available studies that performed prospective screening for subclinical leaflet thrombosis.

**Study**	**Year**	**N**	**Imaging**	**Incidence**	**Time to CT**	**Hemodynamic consequence**	**Antithrombotic treatment**	**Predictors**	**Clinical outcome**
Makkar et al. (2)	2015	55	CT	TAVR-HAM: 40%	1 month	No difference in gradient	Warfarin vs. DAPT: 0% vs. 51% (*P =* 0.007)	Absence of OAC	No significant differences in TIA, stroke, death
Hansson et al. (3)	2016	405	CT	TAVR-HALT: 7%	1–3 months	Significantly higher gradient at 3 months: 10 vs. 8 mmHg; *P =* 0.003	Warfarin vs. no warfarin: 1.8 vs. 10.7% (RR 6.1; 95% CI 1.9–19.8)	Larger THV size; absence of warfarin; absence of AF; GFR < 30 ml/min	5/28 developed obstructive CVT with heart failure symptoms; no cases of CVT in group without HALT
Pache et al. (4)	2016	156	CT	TAVR-HALT: 10%	5 days	Significantly higher gradient: 15 vs. 12 mmHg; *P =* 0.026	No significant difference between OAC vs. SAPT vs. DAPT	No significant predictors	At 2–6 months: no stroke, no bleeding, 1 non-CV death.
Chakravarty et al. (5)	2017	890	CT	TAVR-HAM: 13% SAVR-HAM: 4%	1–12 months	Mean gradient > 20 mmHg and increase > 10 mmHg in case of HAM vs. without HAM: 16 vs. 6 and 15 vs. 1%; *P <* 0.001	OAC vs. DAPT: 4 vs. 15%; *P <* 0.001; OAC vs. SAPT: 4 vs. 16%, *P <* 0.001; DAPT vs. SAPT: 15 vs. 15%, *P =* 0.83	Higher in TAVR vs. SAVR (*P =* 0.001).	Similar rate of all stroke in HAM vs. no HAM(6 vs. 3%, *P =* 0.10), but higher rate of TIA in HAM vs. no HAM (6 vs. 1%, *P =* 0.0005).
Marwan et al. (6)	2017	78	CT	TAVR-HALT/HAM: 23%	4 months	No significant differences in mean gradient; in symptomatic pts mean gradient significantly higher than in asymptomatic pts: 30 vs. 12 mmHg, *P =* 0.002.	More pts on OAC in non-HAM vs. in HAM group (50 vs. 11%, *P =* 0.02)	Absence of OAC.	No TIA/stroke or thromboembolism at 12 months
Vollema et al. (7)	2017	434	TTE + CT	TAVR-HALT/HAM: 12%	1–36 months	Only 1 of CT+ pts had increased mean gradient.	N/A	N/A	No significant differences in TIA, stroke.
Yanagisawa et al. (8)	2017	70	CT	TAVR-HALT/HAM:discharge: 1% 6 m: 10% 1 y: 14%	within 3 days, at 6 m and 12 m	HALT was not associated with increased mean gradient (*P =* 0.41)	3/10 with HALT received anticoagulation	Males; larger SOV; larger THV size	No significant differences in stroke or all-cause death.
Dalén et al. (10)	2017	47	CT	SAVR-HALT: 38% SAVR-HAM: 28%	1–36 months	N/A	HALT: 5/18 (28%) HAM: 3/13 (23%) treated with (N)OAC	No significant predictors	3/47 perioperative stroke; 1/17 TIA in FU (406 d): 2 with vs. 2 without HALT/HAM
Ruile et al. (9)	2018	754	CT	TAVR-HALT/HAM: 16%	5 days	No difference in gradient.	Non-significant difference for anticoagulation	N/A	No significant differences in TIA/stroke or all-cause mortality. Hemodynamic valve deterioration: 3 pts (2.5%) with HALT vs. 5 pts (0.8%) without HALT; *P =* 0.006.

*(N)OAC, (novel) oral anticoagulant; AF, atrial fibrillation; CI, confidence interval; CT, computed tomography; CV, cardiovascular; CVT, clinical valve thrombosis; DAPT, double antiplatelet therapy; GFR, glomerular filtration rate; HALT, hypo-attenuating leaflet thickening; HAM, hypo-attenuation affecting motion; N/A, not available; RR, relative risk; SAPT, single antiplatelet therapy; SAVR, surgical aortic valve replacement; SLT, subclinical leaflet thrombosis; SOV, sinus of Valsalva; TAVR, transcatheter aortic valve replacement; THV, transcatheter heart valve; TIA, transient ischemic attack; TTE, transthoracic echocardiography*.

## Diagnosis

The diagnostic sequence is fundamentally different for clinical valve thrombosis and subclinical leaflet thrombosis. As embedded in the terminology, “clinical” valve thrombosis presents most of the time with clinical symptoms, whereas “subclinical” leaflet thrombosis typically presents as an incidental finding on MDCT or TEE imaging in asymptomatic patients.

European guidelines recommend echocardiography, including measurement of transprosthetic gradient, at baseline (within 30 days), at 1 year after valve implantation and annually thereafter, and earlier if any new symptom occurs (18). However, first-line screening with TTE for prosthetic valve dysfunction has been reported to have low sensitivity. Therefore, additional TEE imaging is recommended in case of clinical suspicion of prosthetic valve dysfunction.

A predictive model for surgical bioprosthetic heart valve thrombosis has been proposed, combining three echocardiographic and two clinical predictors: (1) presence of >50% increase in mean transvalvular gradient, (2) increase of cusp thickness, (3) abnormal cusp mobility, and (4) presence of paroxysmal atrial fibrillation with (5) sub-therapeutic international normalized ratio (INR) under warfarin therapy were reported to be predictors of bioprosthetic valve thrombosis (19).

In clinical valve thrombosis, symptoms of heart failure may appear progressively or even (sub)acutely, or the diagnosis may be made following a left-sided thromboembolic event. In such patients with symptoms and/or a sudden increase of transprosthetic gradient or new central aortic regurgitation, an additional TEE should be performed to examine other causes of THV deterioration. MDCT may be considered whenever TEE is not sufficient for clear diagnosis. However, in two retrospective observational studies, only a minority of TAVR patients (26–39%) with THV dysfunction and presence of valve thrombosis, presented with dyspnea (15, 16). This demonstrates that diagnosis is often established based on echocardiographic findings, even before symptoms appear. In these studies, transvalvular aortic gradients were elevated–with more than 90% of patients having a mean gradient >20 mmHg–irrespective of the presence of symptoms, with most often appearance of thickened leaflets or thrombotic apposition, and in a minority of cases a visible thrombotic mass as seen by TTE (15).

The functional consequence may be isolated valvular stenosis, a combined valvular stenosis-regurgitation, or pure valvular regurgitation as reported for surgical bioprosthesis (19). Accordingly, one other study involving TAVR patients reported a significant increase of NT-proBNP levels in patients with clinical valve thrombosis (16). In these studies, clinical valve thrombosis was detected between 3 weeks and 1 year after valve implantation, indicating that this phenomenon is not only limited to the first 3 months following AVR (15, 16).

In case of subclinical leaflet thrombosis, where patients are asymptomatic, diagnosis is often made incidentally on cardiac MDCT-imaging, as part of a clinical study protocol. Changes in transvalvular gradient are more subtle and often still within normal range (2, 4, 5). In the combined SAVORY/RESOLVE registry, including both TAVR and SAVR patients, the mean aortic transvalvular gradient in the HAM-group was 13.8 ± 10.0 mmHg as compared to 10.4 ± 6.3 mmHg in patients with normal leaflet motion (5). Three studies have performed sequential imaging over time, and all of these showed that HALT and HAM can either remain stable, worsen or even regress over time (8, 9, 20). Two of these studies reported that OAC protects against progression (9, 20). Other studies did not show any association between “time to CT” and the presence of subclinical leaflet thrombosis (5, 6). Importantly, all studies demonstrated that subclinical leaflet thrombosis may appear both early or late after valve implantation, with varying evolutions of either progression, stability or regression at different time intervals, and diverge among different antithrombotic strategies.

TEE has shown to be equally sensitive for the detection of leaflet thickening, thrombotic appositions, or restricted leaflet mobility as compared to MDCT imaging (2). However, as the clinical significance of subclinical leaflet thrombosis is not clear, and MDCT requires exposure to radiation and contrast agent and TEE is limited by its rather invasive character, it is not recommended to use MDCT-imaging or TEE as a routine post-procedure screening tool for subclinical valve thrombosis outside clinical studies.

## Predisposing Factors and Pathophysiology

Little is known about the complex pathophysiology of bioprosthetic valve thrombosis and most investigations are based on imaging studies. The difference in incidence of prosthetic valve thrombosis for surgical and transcatheter aortic bioprosthesis also suggests the involvement of multiple and divergent pathophysiological mechanisms, including device variables, host variables, and antithrombotic therapy.

### Device Variables

Different devices have different valve designs, consist of different materials and use different implantation techniques, all of which may influence the mechanism and risk for thrombus formation. In contrast with SAVR, the native aortic valve leaflets remain present in TAVR and are pushed aside in the sinus of Valsalva. This may not only cause mechanical differences with differing valve geometry and hemodynamics, the remaining (damaged) native valve tissue may also induce thrombosis due to exposure of tissue factor. Besides this, contact with the artificial surface of the bioprosthesis may promote thrombus formation through complex cascades beginning with plasma protein absorption to fibrinogen, subsequent platelet, leucocyte, and red cell adhesion, thrombin generation, and complement activation (21). Pathology studies show that the initial fibrin coat on the prosthetic surface is replaced by neointima within ~3 months (22). However, delayed endothelialisation, damage of leaflet tissue by THV crimping or post-dilatation, and differences in type of leaflet tissue may influence thrombogenicity of this surface. Moreover, one study showed that porcine surgical bioprostheses are at higher risk for valve thrombosis as compared to bovine tissue (23), whereas one other study reported that residual presence of aldehydes–a substance contained in the storage liquid for storage of these bioprostheses–may also contribute to leaflet thrombosis in case of insufficient cleansing (24).

Also flow dynamics differ according to valve design and can be influenced by the potential occurrence of regional malappositioning or underexpansion of THVs. Areas with turbulence, such as the neo-sinuses (created after TAVR), may lead to increased “shear stress” causing endothelial damage and may also create sites with “low-flow,” thereby increasing the risk of thrombosis. In addition, it has been reported that the THV stent frame geometry may be important in the process of subclinical leaflet thrombosis (25). Regional underexpansion of the THV stent frame has been associated with an increased incidence of subclinical leaflet thrombosis, particularly for THVs with an intra-annular valve position. The hypothesis is that, in case of THV underexpansion, the leaflet may not completely unfold and be more prone to thrombus formation. In one other study, intra-annular THVs and deeper implantation of supra-annular self-expanding THVs resulted in larger neo-sinuses and, hence, a higher risk for leaflet thrombosis (26). Consequently, it has been suggested that bicuspid valves may be associated with a higher risk for leaflet thrombosis, as THVs end up more often non-circular—hence, underexpanded—and bicuspid anatomies are sometimes linked with a larger sinus of Valsalva. However, evidence confirming this theoretical link is still missing. According to a recent meta-analysis that pooled data from seven observational studies, also here THVs with intra-annular valve design were associated with a higher risk for leaflet thrombosis as compared to THVs with supra-annular valve design (27). On the other hand, an *in-vitro* study had generated the hypothesis that post-dilatation may increase the risk for leaflet thrombosis because of histologically proven tissue damage to the leaflets. However, no clinical studies have confirmed this relation and even a lower rate of leaflet thrombosis following post-dilatation has been reported (25).

Host variables may potentially also predispose for device thrombosis. Patient-specific co-morbidities that are known to provoke a pro-thrombotic state are renal insufficiency, diabetes mellitus, heart failure, atrial fibrillation, chronic anemia, smoking, etc. These characteristics may also play an important and contributing role in the incidence of valve/leaflet thrombosis.

Finally, the chosen antithrombotic strategy after AVR has been reported to have a major influence on the development of both clinical valve thrombosis and subclinical leaflet thrombosis. In two retrospective trials, a significantly lower rate of clinical valve thrombosis was reported for TAVR patients on OAC therapy as compared to patients on APT therapy (15, 16). Other studies also showed a significantly lower rate of subclinical leaflet thrombosis in patients on OAC, while it appears there is no significant difference in its incidence for patients on single vs. double APT (2–6).

## Clinical Consequences

In clinical valve thrombosis, the functional consequence might be isolated valvular stenosis, a combined valvular stenosis/regurgitation, or pure valvular regurgitation, leading to symptoms of dyspnea. These symptoms may occur progressively over time, but in some cases, may also lead to a more (sub)acute setting of heart failure. Another apparent risk in case of clinical valve thrombosis is the risk for thromboembolic events, which can present as a transient ischemic attack (TIA), stroke, or peripheral embolism (11, 12).

In subclinical leaflet thrombosis, patients are per definition asymptomatic. There is, however, a concern that subclinical leaflet thrombosis might progress toward clinical valve thrombosis, increase the risk for thromboembolic events or impair the durability of the aortic bioprosthesis.

Regarding the association between subclinical leaflet thrombosis and stroke/TIA, there have been reports raising some concerns. In the SAVORY/RESOLVE registry, HAM was associated with an increased incidence of TIA (5). However, this finding needs to be interpreted with caution since the status of the leaflet was not known at the moment of TIA, with observation of long temporal separation between the clinical event and the MDCT scan (28). Even more, studies have shown natural temporal dynamic changes between HALT, HAM, and normal status without changing antithrombotic regimen (8, 9, 20). In contrast, a prospective trial including 434 TAVR patients studied with MDCT or echocardiography did not show any increased stroke risk at 3 years of follow-up in patients with subclinical leaflet thrombosis. The overall stroke rate in this latter study was 3.2%, but none of the strokes were related with subclinical leaflet thrombosis, nor did they have differences in valve hemodynamics (7). Accordingly, despite the fact that subclinical leaflet thrombosis has higher incidence in TAVR as compared to TAVR, cerebrovascular event rates at short and mid-term follow-up are not higher after TAVR as compared to SAVR (1, 29–31). This does not further support this suggested link between subclinical leaflet thrombosis and an increased risk of neurological events.

Secondly, concerns have been raised about the possible negative impact of subclinical leaflet thrombosis on long-term valve durability. A recent retrospective analysis showed that absence of OAC was independently associated with an increase in transvalvular gradient at long-term follow-up (32). However, the fact that this phenomenon is a dynamic process makes it hard to investigate its possible impact on long-term durability. This assumption is not supported by mid-term data demonstrating THV durability to be non-inferior as compared to surgical bioprosthetic valves, although subclinical leaflet thrombosis occurs more frequent after TAVR than after SAVR (29, 30, 33).

Finally, it is still not clear whether subclinical leaflet thrombosis forms a substrate or is the biological precursor of clinical valve thrombosis. In a large prospective study, encompassing 754 TAVR patients that underwent CT-imaging, reporting an incidence of subclinical leaflet thrombosis of 16%, there was no increase in stroke or mortality at a mean follow-up time of 406 days for those patients with subclinical leaflet thrombosis. However, there was a significantly higher rate of patients that developed clinical valve thrombosis in the leaflet thrombosis-group as compared to the non-leaflet thrombosis-group (2.5 vs. 0.8%; *P* = 0.006) (34). In another study, five out of 28 TAVR patients with subclinical leaflet thrombosis developed obstructive valve thrombosis associated with heart failure symptoms, while this did not occur in the group of patients without leaflet thrombosis (3). Whether there is a relationship between these two phenomena is not clear for the time being, but it may influence the strategy of follow-up and treatment in patients diagnosed with subclinical leaflet thrombosis.

## Antithrombotic Strategy

**Figure 3:**
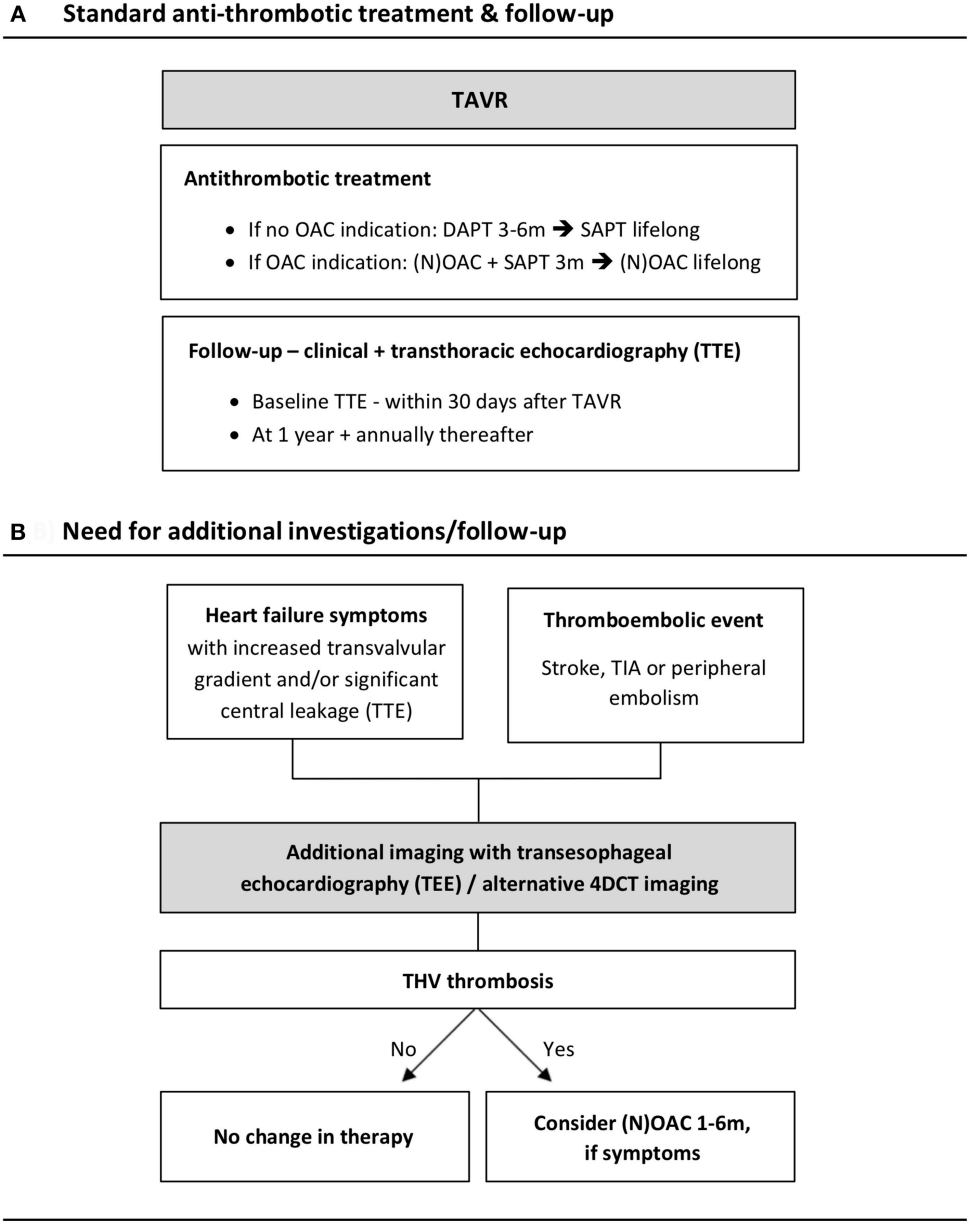
Algorithm for the follow-up of TAVR patients. **(A)** Standard antithrombotic treatment and follow-up according to the European guidelines. **(B)** Need for additional investigations/follow-up in case of heart failure symptoms and/or thromboembolic events. 4DCT, 4-dimensional computed tomography; DAPT, double antiplatelet therapy; (N)OAC, (novel) oral anticoagulant; SAPT, single antiplatelet therapy; TAVR, transcatheter aortic valve replacement; TEE, transesophageal echocardiography; THV, transcatheter heart valve; TIA, transient ischemic attack; TTE, transthoracic echocardiography.

As a standard therapy, both the European (EU) and American (US) guidelines recommend DAPT for the first 3 to 6 months following TAVR in case of no other OAC indication (18, 35). The US guidelines also recommend to consider treatment with OAC during the first 3 months after bioprosthetic valve implantation, in patients at low bleeding risk (Class IIb, level of evidence C). For patients with atrial fibrillation, US guidelines make no specific recommendation, while EU guidelines recommend a combination of vitamin K-antagonist (VKA) and aspirin or thienopyridine, considering the bleeding risk of an individual patient (Figure 3). OAC therapy has shown to prevent the development of both clinical valve thrombosis and subclinical leaflet thrombosis, while APT does not show this effect (2, 3, 5, 16). Accordingly, treatment with OAC seems–at least temporarily–to restore leaflet motion in case of HAM.

Multiple ongoing trials, with or without MDCT imaging for leaflet thrombosis, are examining different antithrombotic strategies (Table 2). Interestingly, the randomized ARTE trial came up with evidence supporting aspirin monotherapy above DAPT following TAVR using the Sapien XT THV (Edwards Lifesciences, Irvine, US). Patients in the SAPT-group experienced fewer major and life-threatening bleedings, without increasing the rate of stroke, MACE or death, as compared to the DAPT group (36). Moreover, the randomized GALILEO trial comparing rivaroxaban plus aspirin with standard APT therapy following TAVR was stopped prematurely during the follow-up period due to higher rates of bleeding and death in the rivaroxaban group (*data not published yet*). While waiting for results from other ongoing trials, current data do not support OAC as a standard regimen for all TAVR patients given the increased bleeding risks associated with long-term OAC therapy in this often frail and elderly patient population.

**Table 2:** Overview of completed and ongoing trials investigating different antithrombotic strategies after TAVR.

**Trial**	**Anti-thrombotic strategy**	**Target population**	**Study population**	**Anticipated completion date**	**Outcome**
Warfarin and antiplatelet therapy vs. warfarin alone for treating patients with atrial fibrillation undergoing TAVR	VKA vs. VKA + single or double APT	Patients with atrial fibrillation	621	Completed	Similar rate of MACE, stroke and death; but significantly higher rate of major or life-threatening bleedings in VKA + APT group (13 months FU)
ARTE NCT01559298	ASA + clopidogrel vs. ASA alone	Patients without need for chronic OAC	222	Completed	No differences in hemodynamics, MACE, TIA, stroke, and death; less major or life-threatening events in ASA alone group (3 months FU)
GALILEO NCT02556203	Rivaroxaban 10 mg + ASA (3 months), followed by rivaroxaban alone vs. DAPT (3 months), followed by ASA alone	Patients without need for chronic OAC	1644	2018	Stopped prematurely because of increased bleeding and mortality in the rivaroxaban group.
AUREA NCT01642134	ASA + clopidogrel vs. VKA	Patients without need for chronic OAC	124	2019	–
POPular–TAVI NCT02247128	ASA + clopidogrel vs. ASA alone, or OAC + clopidogrel vs. OAC alone	All-comers	1,000	2020	–
ATLANTIS NCT02664649	Apixaban vs. standard of care (VKA or SAPT/DAPT)	All-comers	1,510	2020	–
AVATAR NCT02735902	ASA + VKA vs. VKA alone	Patients with underlying indication for chronic OAC	170	2020	–
ENVISAGE-TAVI AF NCT02943785	VKA vs. edoxaban (both with APT if needed)	Patients with atrial fibrillation	1,400	2020	–
ADAPT-TAVR NCT03284827	Edoxaban vs. ASA + clopidogrel (min. 6 months)	Patients without absolute indication for chronic OAC	220	2020	–

*APT, antiplatelet therapy; ASA, acetylsalicylic acid; DAPT, double antiplatelet therapy; FU, follow-up; MACE, major adverse cardiovascular events; OAC, oral anticoagulant; SAPT, single antiplatelet therapy; TIA, transient ischemic attack; VKA, vitamin-K antagonist*.

For patients with clinical valve thrombosis, two studies reported a beneficial evolution of the transprosthetic gradients following VKA treatment (15, 16). However, in one study relapse occurred after stopping VKA, while this was not observed among patients that were switched from VKA to non-VKA oral anticoagulants (16). In case the diagnosis of clinical valve thrombosis is confirmed, VKA therapy should be started if no contraindication is present. Optimal type and duration of OAC in such cases is still unclear, but it should at least be continued until the thrombus has resolved and the valve function is restored. Currently there is no evidence supporting prolongation of OAC therapy after restoration of the normal valve function, although careful clinical and echocardiographic follow-up is warranted. In case of recurrent clinical valve thrombosis, prolongation of OAC therapy could be considered, however, in order to take such decision, one should carefully consider the patient's bleeding risk.

Similarly, almost all patients receiving OAC for subclinical leaflet thrombosis showed full resolution of leaflet attenuations and restoration of normal leaflet motion (4, 5). In one study, relapse occurred in half of the patients when OAC was interrupted (2). Accordingly, progression from HALT to HAM never occurred in patients on OAC, but was reported in 13/60 (22%) patients on APT (20). In one other study comprising 51 patients with HALT, control MDCT-scan after a median of 86 days showed regression in all of the 22 patients on OAC, while 11 of 29 patients on APT had progression of leaflet thickening. After a median of 91 days after discontinuation of anticoagulation, MDCT performed in ten patients revealed a significant increase in leaflet restriction and thickness, indicating that treatment of this condition may be challenging (9).

In summary, the current knowledge does not support routine post-procedural MDCT in order to screen for subclinical leaflet thrombosis, because of additional exposure to radiation and contrast agent as well as the lack of evidence on the impact of OAC therapy on long term outcomes. In case subclinical leaflet thrombosis is detected, it may be reasonable to shorten the echocardiographic follow-up interval and tailor the antithrombotic therapy to each individual patient in case of increasing transprosthetic gradient or thrombo-embolic event (Table 3).

**Table 3:** Bioprosthetic valve thrombosis: clinical presentation, diagnosis, and treatment.

	**Clinical valve thrombosis**	**Subclinical leaflet thrombosis**
Clinical presentation	Dyspnea Heart failure symptoms Left sided thromboembolic event (TIA, stroke, or peripheral embolism)	Asymptomatic No routine screening (MDCT, TEE)
Diagnosis	TTE/TEE Leaflet thickening Thrombotic apposition(s) Thrombotic mass Increased transvalvular gradient	TEE/MDCT Leaflet thickening Leaflet motion impairment
Treatment	If stable patient: Initiate 6–12 week OAC Echocardiographic follow-up Restart (N)OAC if re-occurrence Consider redo-AVR if no effect of anticoagulation If unstable patient: Redo-AVR, if possible VIV-TAVR	Sharpened vigilance No routine (N)OAC Shorten echocardiographic follow-up interval and consider (N)OAC in case of increasing transvalvular gradient or thromboembolic event

*AVR, aortic valve replacement; MDCT, multi-detector computed tomography; OAC, oral anticoagulant; TEE, transesophageal echocardiography; TIA, transient ischemic attack; TTE, transthoracic echocardiography; VIV, valve-in-valve*.

## Conclusion

Bioprosthetic valve thrombosis encompasses two different entities: clinical valve thrombosis and subclinical leaflet thrombosis. Patients with clinical valve thrombosis often present with heart failure symptoms and an increase in transprosthetic gradient, while subclinical leaflet thrombosis is an incidental finding on post-procedural TEE and/or MDCT imaging. Treatment with OAC is recommended for clinical valve thrombosis, although the optimal medical treatment and duration is not clear yet. Whether subclinical leaflet thrombosis is associated with an increased risk for thromboembolism or accelerated valve degeneration is still a matter of speculation. Based on current evidence, it is not recommended to perform routine TEE/MDCT to screen for subclinical leaflet thrombosis, nor to treat with OAC in detected cases. However, it is recommended to shorten the echocardiographic follow-up interval in case of subclinical leaflet thrombosis. Ongoing and future randomized trials are expected to contribute to a better and more patient-tailored antithrombotic therapy following AVR with a bioprosthetic heart valve.

## Author Contributions

LR, LS, and ODB all contributed to the conception and design of this review. LR wrote the first draft of the manuscript and was extensively reviewed by LS and ODB. All authors have approved the final submitted version.

### Conflict of Interest Statement

The authors declare that the research was conducted in the absence of any commercial or financial relationships that could be construed as a potential conflict of interest.
